# Expression of the transcription factor CTCF in invasive breast cancer: a candidate gene located at 16q22.1

**DOI:** 10.1038/sj.bjc.6602144

**Published:** 2004-08-31

**Authors:** E A Rakha, S E Pinder, C E Paish, I O Ellis

**Affiliations:** 1 The Breast Unit, Department of Histopathology, Nottingham City Hospital NHS Trust, Hucknall Road, University of Nottingham, Nottingham NG5 1PB, UK

**Keywords:** breast cancer, chromosome 16, CTCF, immunohistochemistry

## Abstract

CTCF is a ubiquitous 11-zinc-finger protein that plays a role in gene silencing or activation, chromatin insulation and genomic imprinting. The CTCF gene has been mapped to the chromosome band 16q22.1 that shows frequent loss of heterozygosity in breast cancer. The E-cadherin gene is the known tumour suppressor gene (TSG) at this region in lobular carcinomas; however, the target gene in the more frequent ductal tumours is still unknown. Since CTCF targets include TSGs and oncogenes and it has the ability to inhibit cell growth and proliferation, it has been suggested that it may be the target gene at the 16q22.1 in ductal carcinomas. In the present study, tissue microarray technology was used to study the expression pattern of CTCF immunohistochemically in 344 cases of invasive breast carcinoma and its expression was correlated with clinicopathological variables and patient outcome. Results showed that breast tissues express CTCF in the parenchymal cells of the normal ducts and lobules but with a variable percentage of positive cells. Staining of CTCF was detected in the nuclei and cytoplasm of the malignant cells, but no significant loss or decrease of expression was noticed in association with any specific tumour type. There was a significant correlation between expression of CTCF and histological grades; lower expression was associated with grade 3 tumours. Cytoplasmic expression was associated with increased tumour size and with the presence of vascular invasion. However, no association was found between CTCF expression and tumour type, lymph node stage, oestrogen receptor expression or patient outcome. In conclusion, the current results show that CTCF, although it may play a role in breast carcinogenesis, is unlikely to be the TSG targeted by the 16q22.1 loss in breast cancer and thus another gene or genes at this region remain to be identified.

Loss of heterozygosity (LOH) at the long arm of chromosome 16 (16q) is one of the most frequent somatic genetic events in both lobular and ductal carcinomas of the breast (Skirnisdottir *et al*, 1995; Cleton-Jansen *et al*, 2001). The smallest region of overlap at 16q in breast cancer has been located at 16q22.1 (Frengen *et al*, 2000), indicating the presence of a tumour suppressor gene (*TSG*) at this region. In the search for the target gene, E-cadherin has been proven to be the TSG by the identification of gene mutations and loss of protein expression, but only in the lobular type carcinomas (Kanai *et al*, 1994; Berx *et al*, 1995; Berx *et al*, 1998; Acs *et al*, 2001). Thus, another TSG (s) is the target of 16q22.1 LOH in the more frequent ductal carcinomas that remains to be identified. Any gene located in the vicinity of 16q22.1 could be considered as a candidate gene and should be examined to assess its role in breast cancer.

The CTCF gene has been mapped to 16q22.1 (Filippova *et al*, 1998). It encodes a DNA-binding 11-zinc-finger protein that shows a highly versatile function and multiple DNA sequence specificity (Filippova *et al*, 1996, 1998). CTCF is a widely expressed transcription factor that is involved in different aspects of gene regulation including promoter activation (Vostrov and Quitschke, 1997) and repression (Filippova *et al*, 1996), hormone-responsive gene silencing (Burcin *et al*, 1997), methylation-dependent chromatin insulation and genomic imprinting (Kanduri *et al*, 2000; Filippova *et al*, 2002). In addition, it has been demonstrated that CTCF can inhibit cell growth and induce cell cycle arrest at multiple stages (Rasko *et al*, 2001).

The tumour suppressor role of CTCF was suspected because of its involvement in regulating the expression of some genes that are directly implicated in cancer (i.e., *MYC, IGF2, p53, P27, p19/ARF and BRCA1*) (Bell and Felsenfeld, 2000; Klenova *et al*, 2002; Qi *et al*, 2003), its cell growth inhibitory effect and its genetic mapping to 16q22.1. In addition, some tumour-specific mutations have been detected in some tumours including breast cancer (Ohlsson *et al*, 2001; Filippova *et al*, 2002). The role of CTCF in cancer has previously been studied in some human tumours (Takai *et al*, 2001; Filippova *et al*, 2002; Yeh *et al*, 2002; Aulmann *et al*, 2003; Ulaner *et al*, 2003); however, its role in breast cancer is still unclear.

If CTCF was the target gene of 16q22.1 LOH in ductal carcinoma of the breast, then we would predict that a large proportion of ductal tumours might show marked or complete loss of its expression compared with other histological types and/or with the normal parenchymal tissues. Thus, in the present study, protein expression of CTCF was examined immunohistochemically in a large series to assess its possible role in invasive breast cancer.

## MATERIALS AND METHODS

### Patients and tumours

Formalin-fixed, paraffin-embedded tissue blocks were obtained from 344 cases of invasive breast carcinoma available from the Nottingham Tenovus Primary Breast Carcinoma Series. This is a well-characterised series of primary operable invasive breast cancer that has been previously used to study a wide range of proteins including, recently, the CD59 protein (Madjd *et al*, 2003). Patient's clinical history and tumour characteristics including tumour type (Ellis *et al*, 1992), size, histological grade (Elston and Ellis, 1991), lymph node stage, Nottingham Prognostic Index (NPI) and oestrogen receptor expression were obtained from the database. Patients had a median age of 53 years (range 18–85 years). The tumours were as follows: 29 cases were pure lobular (8.4%), 32 mixed lobular and ductal (9.3%), 214 ductal/no special type (NST) (62.2%), five mixed NST and special type (1.5%), 12 tubular (3.5%), 42 tubular mixed (12.2%) and 10 cases miscellaneous tumour types (2.9%). Survival data including survival time, disease-free interval, development of distant metastasis and recurrence was available for 294 patients. The median follow-up time was 49.5 months (range 5–78 months). The NPI was calculated using the following equation: NPI=0.2 tumour size (cm)+grade (1–3)+lymph node score (1–3) (Galea *et al*, 1992). This index predicts the survival of patients with invasive breast cancer and may be divided into three groups: good prognosis (⩽3.4), moderate prognosis (3.41–5.4) and poor prognosis group (>5.4) (Kollias *et al*, 1999).

### Tissue arrays and immunohistochemistry

Tumour samples were arrayed as previously described (Kononen *et al*, 1998). The tissue microarray blocks were constructed in three copies, each containing one sample from a different region of the tumour. In addition, whole tumour tissue blocks from 40 cases were stained and used to examine CTCF expression in normal breast tissues. Immunohistochemical staining was performed in accordance with standard procedures on 4 *μ*m-thick sections. Briefly, tissue slides were deparaffinised with xylene and then rehydrated through three changes of alcohol. Endogenous peroxidase activity was blocked by incubation in a 0.3% hydrogen peroxide/methanol buffer. Antigen retrieval was carried out by microwave treatment of the slides in sodium citrate buffer (pH 6.0) for 20 min. Then, the slides were rinsed in Tris-buffered saline (TBS) (pH 7.6) and incubated with normal swine serum (NSS) in TBS (1 : 5) to block nonspecific staining. The slides were then incubated overnight with a goat polyclonal anti-CTCF antibody (Santa Cruz Biotechnology, sc-5916) and used in an optimal dilution of 1 : 100 (v v^−1^ in NSS/TBS). After washing with TBS, sections were incubated with the biotin-labelled secondary antibody (1 : 100) for 30 min, and then avidin–biotin complex (1 : 100) for a further 45 min. 3-3′Diaminobenzidine tetrahydrochloride was used as a chromogen and the sections were counterstained with Mayer's haematoxylin.

**Figure 1:**
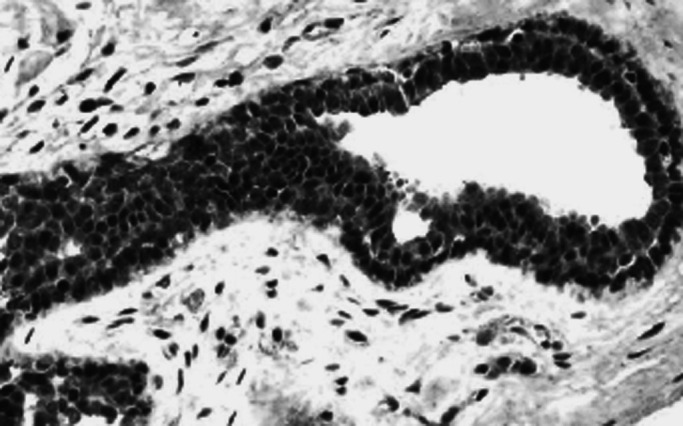
Expression of CTCF in the normal parenchymal cells of the breast (dilution of the primary antibody was 1 : 50, overnight).

To verify the specificity of the CTCF immunoreactivity, blocking studies were performed by preincubation of the primary antibody (at different dilutions) for 2 h at room temperature with the protein fragment used to raise it (Santa Cruz Biotechnology, sc-5916 P) prior to its application to the tissues according to the supplier data sheet protocol. This abolished staining of the antibody (Figures 1

and 2

**Figure 2:**
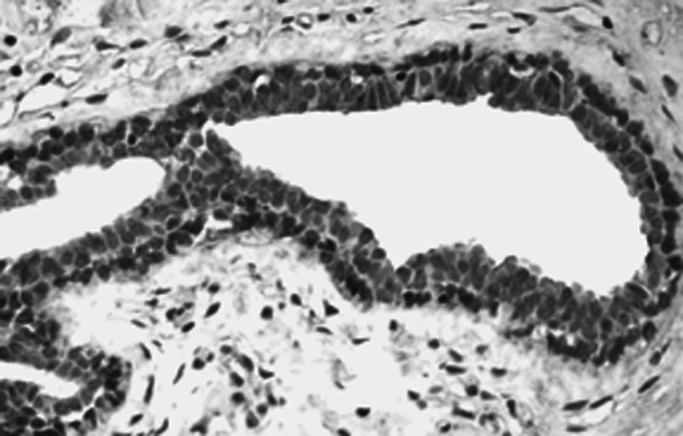
The same case after peptide blocking of the antibody showing marked reduction of staining.

). Preincubation with equivalent concentration of an irrelevant peptide had no effect on immunostaining. To further confirm the subcellular localisation o the CTCF protein, another primary antibody was used; a rabbit polyclonal anti-CTCF antibody (AbCam Ltd, Cambridge, UK, ab 10571) in a dilution of 1 : 20 for 2 h incubation using the same staining conditions as mentioned above. Negative control sections with omission of the primary antibody were included and were consistently negative. Tonsil and normal breast tissues were used as positive controls.

### Evaluation of immunohistochemical staining

CTCF staining was evaluated separately in the nuclei and cytoplasm of the malignant cells. Assessment of the staining results was based on a semiquantitative approach. A modified histochemical score (H-score) was used for assessment (McCarty *et al*, 1985). This includes a determination of both intensity of staining and percentage of stained cells. For the intensity, a score index of 0, 1, 2 and 3 corresponding to absent, weak, moderate and strong staining intensity was used and the percentage of positive cells at each intensity was estimated. A final score of 0–300 was the product of both the intensity and the percentage. Cutoff points of expression was determined according to the histogram distribution of the final scores. Two cores were evaluated from each tumour. Each core was scored individually, and then the mean of the two readings was calculated (Camp *et al*, 2000). The cases were scored without knowledge of patient data by one observer on two separate occasions and a good correlation between the results was found.

### Statistical analysis

Statistical analysis was performed using SPSS 10.0 statistical software. Associations between clinicopathological variables and expression of CTCF were analysed using the *χ*^2^ and Spearman rank correlation tests. Correlation between protein expression levels and overall survival and disease-free interval were analysed using Kaplan–Meier curves with the differences estimated using the Mantel–Cox log-rank test. A *P*-value of <0.05 was considered significant.

## RESULTS

### CTCF expression in the breast

**Figure 3:**
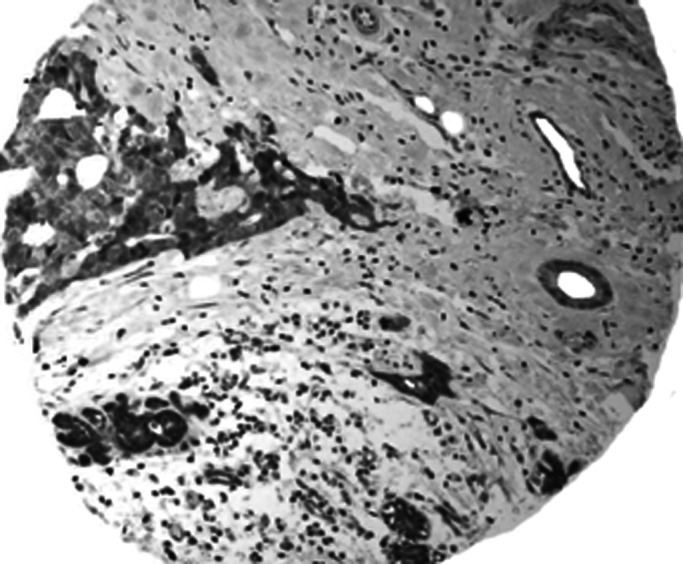
TMA showing expression of CTCF in the normal and malignant tissues of the breast.

Results showed that breast tissues express CTCF in the parenchymal cells of the normal ducts and lobules but with a variable percentage of positive cells. The staining pattern of the normal tissue was mainly nuclear but cytoplasmic staining was also evident in some cells. No membranous staining was detected. Lymphocytes and endothelial cells showed positive staining pattern. Malignant cells showed a heterogeneous nuclear and/or cytoplasmic granular staining pattern (Figures 3

, 4

**Figure 4:**
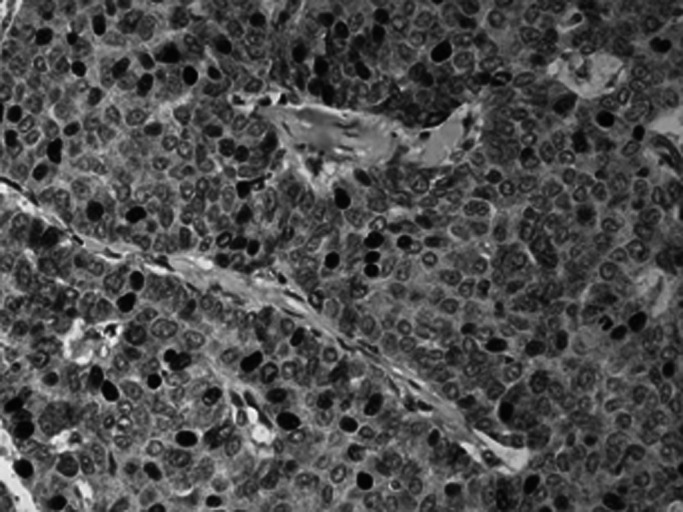
A case of ductal/NST carcinoma showing heterogeneous pattern of positive nuclear staining of CTCF.

, 5

**Figure 5:**
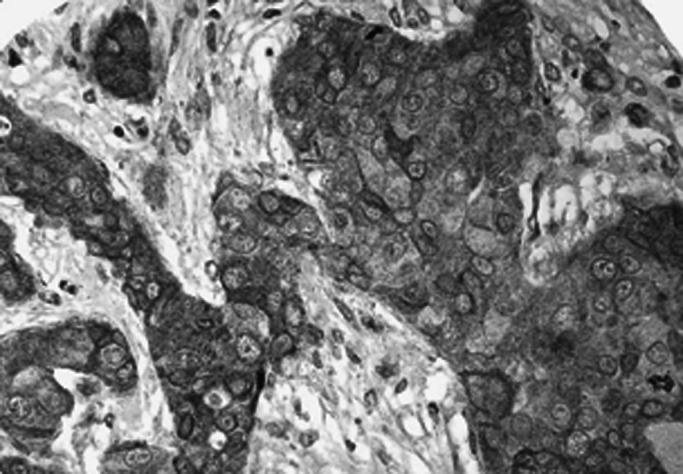
A case with positive cytoplasmic staining.

and 6

**Figure 6:**
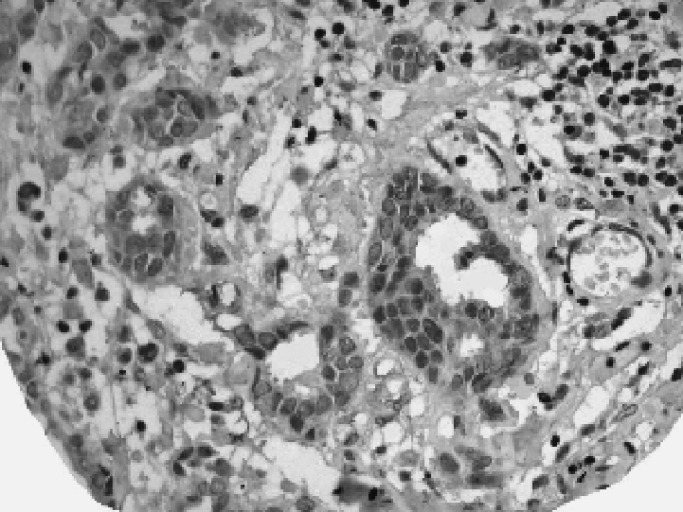
A case with reduced or weak staining of CTCF showing positive staining of stromal cells.

). Normal parenchymal cells in whole tissue sections as well as normal tissue entrapped in the cores were used as internal controls. Only staining of the invasive part of the tumours was considered. Both anti-CTCF antibodies used in this study showed the same pattern of staining and a significant correlation of their staining results was noticed (*P*<0.001).

Cases were classified according to nuclear staining into two groups; reduced (H-score<50), 202 cases (58.7%); and positive (⩾50), 142 cases (41.3%); and according to cytoplasmic staining into; reduced (H-score<100), 79 cases (23%); and positive (⩾50), 265 cases (77%). There was a significant positive correlation between nuclear and cytoplasmic staining of all cases (*r*=0.618, *P*<0.001). The nuclear and cytoplasmic expression of CTCF in the malignant tissue varied between cases with the positive cells varying in intensity and percentage, but no specific pattern of staining or significant loss or decrease of expression was noticed in association with any particular type of breast cancer.

### Correlation between CTCF expression and clinicopathological features in breast carcinoma

Table 1

**Table 1:** Correlation between CTCF expression and clinicopathological features

		CTCF expression					
		**Cytoplasmic**		***χ*^2^**	**Nuclear**		***χ*^2^**
**Variables**	**No**	**Negative**	**Positive**	***p*-value**	**Negative**	**Positive**	***p***-**value**
*1-Age (years)*							
<40	47	15	32		30	17	
				*9.399*			0.684
40–50	103	23	80		60	43	
				*0.024*			0.877
51–60	97	13	84		56	41	
>60	95	28	67		54	41	
							
*2-Tumour type*							
				0.111			0.003
Lobular	61	15	46		36	25	
				0.739			0.959
Nonlobular	283	64	219		166	117	
							
*3-Size (cm)*							
				*4.662*			2.592
⩽2	192	36	156		105	87	
				*0.031*			0.107
>2	150	43	107		95	55	
							
*4-Grade*							
1	53	19	34	*6.933*	27	26	*6.237*
2	123	29	94	*0.031*	65	58	*0.044*
3	168	31	137		110	58	
							
*5-LN status*							
				1.816			0.007
Negative	196	50	146		114	82	
				0.178			0.932
Positive	145	28	117		85	60	
							
*6-Stage*							
1	195	49	146	4.187	113	82	0.564
2	112	18	94	0.123	64	48	0.754
3	31	9	22		20	11	
							
*7-VI*							
				*7.344*			1.063
No or probable	215	60	155		130	85	
				*0.007*			0.302
Definite	126	19	107		69	57	
							
*8-NPI*							
Poor	66	13	53	2.44	41	25	*10.34*
Moderate	180	37	143	0.295	116	64	*0.006*
Good	92	26	66		41	51	
							
*9-DM*				0.009			0.748
No DM	262	51	211	0.923	152	110	0.387
Positive DM	32	6	26		16	16	
							
*10-LR*							
				0.131			0.000
No LR	151	48	204		144	108	
				0.718			1.00
Positive LR	65	9	33		24	18	

Lobular group=pure lobular and lobular mixed; Nonlobular group=ductal (NST), tubular, tubular mixed and other special types; LN=lymph nodes; VI=vascular invasion; NPI= Nottingham Prognostic Index; DM=distant metastasis; LR=local and regional recurrence. Italic=statistically significant.

shows the relationship between nuclear and cytoplasmic expression of CTCF and the different clinicopathological features. No correlation was found between CTCF expression and tumour type divided into two groups; lobular (pure lobular and lobular mixed) and nonlobular group (ductal, tubular, mixed and other special types), or according to its specific histologic type (*χ*^2^=12.295, df=8, *P*>0.138 for nuclear expression and *χ*^2^=5.109, df=8, *P*>0.746 for cytoplasmic expression). A significant association was found between nuclear and cytoplasmic expression and histological tumour grade. There was also a correlation between cytoplasmic staining and age, tumour size and the presence of vascular invasion and between nuclear staining and NPI divided into three groups as described, with greater frequency of positive cases in the good prognostic index group of patients (*P*=0.006). However, there was no association between CTCF expression and lymph node stage, the development of distant metastasis, oestrogen receptor expression (*χ*^2^=1.56, *P*=0.118 for nuclear expression and *χ*^2^=0.39, *P*=0.694 for cytoplasmic expression) or with patient outcome including local recurrence, survival time or disease-free interval (Table 2

**Table 2:** Correlation between CTCF expression and outcome

	**CTCF expression**					
	**Cytoplasmic**			**Nuclear**		
**Variables**	**Log rank**	**df**	***P* value**	**Log rank**	**df**	***P* value**
Survival time	1.39	1	0.238	0.02	1	0.887
Disease-free interval	0.43	1	0.511	0.02	1	0.896

df=degree of freedom.

). No association was found between nuclear expression and age, tumour size or the presence of vascular invasion or between cytoplasmic expression and NPI.

### DISCUSSION

Much effort has gone into evaluating the candidate TSG on 16q in invasive ductal carcinoma of the breast, as well as in other tumours that show frequent LOH of this chromosome arm. However, the relevant genes have not been identified. CTCF is a potential target gene because it has been mapped to the smallest region of overlap at 16q in breast cancer (16q22.1) (Filippova *et al*, 1998). It encodes protein involved in the transcriptional regulation of a wide variety of target genes, several of which have been reported to be involved in carcinogenesis (e.g. c-myc, p53, p27 and IGF2) (Ohlsson *et al*, 2001; Klenova *et al*, 2002). In addition, it has been shown to have a profound growth retardation effect and can inhibit cell growth and proliferation (Rasko *et al*, 2001). In the present study, protein expression of CTCF has been studied immunohistochemically (IHC) to evaluate its possible role in breast cancer as well as to assess its prognostic value.

Our results showed that CTCF is expressed in both the nuclei and cytoplasm of the malignant cells; nuclear expression was detected in 41% of breast cancer cases and cytoplasmic expression in 77%. Immunohistochemicl staining of CTCF in invasive ductal carcinoma of the breast has previously been assessed by Aulmann *et al* (2003), who reported moderate to strong nuclear staining in 94.4% (17 out of 18). However, this high percent of positive staining in their study may be related to the small number of cases.

Previous studies of CTCF using immunofluorescence showed nuclear localisation of its protein (Zhang *et al*, 2004), but no cytoplasmic staining has been observed. Therefore, the cytoplasmic expression detected in our study might be either due to unspecific staining or indicative of mutation affecting the nuclear translocation of CTCF protein or as a result of post-translational modification that is commonly found to attenuate transcription factor (Hunter and Karin, 1992; Klenova *et al*, 2001). To rule out the first possibility, we tested the specificity of the antibody binding using peptide blocking and we tried another anti-CTCF antibody that recognises different epitope and has been used before to study the phosphorylation pattern in the C-terminus region of CTCF (Klenova *et al*, 2001). However, our results confirmed the specificity of the observed staining pattern.

Secondly, if this cytoplasmic expression of CTCF was an indication of mutation or inactivity that has any significant tumour suppressor role in a particular tumour type, we would expect an association between the pattern of expression (nuclear, cytoplasmic or reduced) and the tumour type. However, we could not detect any association between the intensity or pattern of expression of CTCF and tumour type and there was neither marked nor complete loss of expression in any specific type, suggesting that CTCF is not the likely TSG in breast cancer. These results are consistent with those of Yeh *et al* (2002) and Cui *et al* (2001) who could not detect mutations or loss of CTCF mRNA in Wilms' tumours and with those of Aulmann *et al* (2003) who used immunohistochemistry, allelic studies and mutation screen of the CTCF gene and did not find loss of CTCF protein expression in invasive ductal carcinoma of the breast.

In the current study, we detected an association between expression of CTCF and histological grade where a high percentage of low-grade tumours that have a less proliferative activity showed positive nuclear expression, while the high-grade tumours mainly showed reduced or absent expression. We also found a correlation between positive cytoplasmic expression and tumours of smaller size. These results are consistent with the previous reports that showed the ability of CTCF to inhibit cell growth and proliferation (Rasko *et al*, 2001). Although we found a correlation between nuclear expression of CTCF and NPI group and between cytoplasmic expression and vascular invasion, we did not find any significant correlation between its expression and other prognostic markers of breast cancer such as lymph node stage or with patient outcome.

In conclusion, our data indicate that CTCF may have a role in breast cancer progression but it is not likely to be the gene that is targeted by 16q22.1 LOH in breast cancer. Further studies of other genes at this region are required to identify the TSG that is the target of 16q22.1 loss in ductal carcinoma of the breast.
